# Validation of the Farsi version of the revised Adolescent Sleep Hygiene Scale (ASHSr): a cross-sectional study

**DOI:** 10.1186/s12888-017-1578-6

**Published:** 2017-12-28

**Authors:** Azita Chehri, Habibolah Khazaie, Soudabeh Eskandari, Sepideh Khazaie, Edith Holsboer-Trachsler, Serge Brand, Markus Gerber

**Affiliations:** 1 Department of Psychology, Kermanshah Branch, Islamic Azad University, Kermanshah, Iran; 2 Sleep Disorders Research Center, Kermanshah University of Medical Sciences (KUMS), Kermanshah, Iran; 3 University of Basel, Psychiatric Clinics (UKP), Center for Affective, Stress and Sleep Disorders (ZASS), Basel, Switzerland; 4 University of Basel, Department of Sport, Exercise and Health, Division of Sport and Psychosocial Health, St. Jakob-Turm, Birsstrasse 320B, 4052 Basel, Switzerland

**Keywords:** Adolescents, Concurrent validity, Confirmatory factor analysis, Factorial validity, Internal consistency, Iran, Sleep hygiene scale, Adolescent, Test-retest-reliability

## Abstract

**Background:**

Restoring sleep is associated with a broad variety of favorable cognitive, emotional, social and behavioral benefits during the day. This holds particularly true for adolescents, as maturational, social, cognitive, emotional and behavioral changes might unfavorably impact on adolescents’ sleep. Among adolescents, poor sleep hygiene practices are a potentially modifiable risk factor that can be addressed via appropriate interventions. Accordingly, having reliable and valid self-report measures to assess sleep hygiene practices is essential to gauge individual responses to behavioral interventions and evaluate sleep hygiene recommendations. The aim of the present study therefore was to translate and to test the psychometric properties (internal consistency, test-retest reliability, factorial and concurrent validity) of the Farsi/Persian version of the revised version of the Adolescent Sleep Hygiene Scale (ASHSr).

**Method:**

A total of 1013 adolescents (mean age: *M* = 15.4 years; *SD* = 1.2; range: 12–19 years; 42.9% females) completed the ASHSr and the Pittsburgh Sleep Quality Index (PSQI) in their classroom during an official school lesson. Further, 20% completed the ASHSr 6 weeks later to evaluate the test-retest reliability. Cronbach’s alpha coefficients were calculated to examine internal consistency, confirmatory factor analysis (CFA) was used to test factorial validity, whereas concurrent validity and test-retest reliability were examined via correlation analyses.

**Results:**

A first-order confirmatory factor analysis (CFA) corroborated the six-factor structure of the ASHSr, including a physiological, behavioral arousal, cognitive/emotional, daytime sleep, sleep environment, and sleep stability factor. A second-order CFA showed that a higher-order sleep hygiene construct explained sufficient variance in each factor. Cronbach’s alpha values ranged between .71 and .75, correlations for test-retest reliability between .82 and .87. Significant correlations were found between most ASHSr scales and the PSQI indices. However, the magnitude of these correlations was weak.

**Conclusions:**

The Farsi/Persian version of the Adolescent Sleep Hygiene Scale can be used as a reliable and valid tool for evaluation of sleep hygiene practices among Farsi/Persian-speaking adolescents.

## Background

Several cross-sectional studies show that restoring sleep is significantly associated with daytime functioning in terms of favorable cognitive, emotional, behavioral, and social processes. This holds true for infants and toddlers [1–4], preschoolers [5, 6], children [7–12], and adolescents [12, 13]. Longitudinal studies further suggest that poor sleep predicts unfavorable cognitive-emotional, social and behavioral patterns in later life [13–20]. As regards adolescents, research showed that approximately half (45%) of 11–17-year-olds report a sleep problem for at least several nights a week, including difficulty in falling asleep, maintaining sleep stability and waking up early [21]. Not surprisingly, inadequate sleep or inappropriate sleep quality among adolescents are associated with academic [22] and psychological performance problems [23, 24], obesity [25], prehypertension [26] and motor vehicle accidents [27].

Scholars have further highlighted that adolescence is a period of vulnerability for the onset of sleep problems [28]. While it is agreed that adolescents still need 8 to 10 h of sleep per night for optimal daytime functioning, a large portion of adolescents do not accomplish these standards [28–30], which is usually attributed to an interplay between biological, social and behavioral risk factors [28, 29]. For instance, biological changes association with puberty and maturation include circadian and homeostatic components of sleep, which influence the sleep-wake cycle, as well as sleep timing, sleep duration, and sleep architecture [30, 31]. Typically, there is a shift of biological sleep patterns during adolescence toward later bedtimes and waking times, which may lead to a greater gap between sleep duration on school-nights and on weekend-nights [32, 33]. Although there is still overlap between sleep pattern of parents and their offspring [34–36], the above pattern may be reinforced by the efforts of teenagers to become more independent form their parents and to decide more freely about social/leisure activities and bedtimes [37]. Based on the findings of a recent systematic review, Becker et al. [38] underscored the importance of the intra-individual variability of sleep/wake patterns in adolescents, and claimed that both basic and clinical sleep recommendations should not only focus on overall sleep duration and sleep habits, but also address the stability of sleep duration and timing. Additionally, previous studies showed that during adolescence, youngsters tend to increase their screen time [39], light exposure at night [40], engage in more evening activities [41], and are more likely to engage in risk behaviors [42], which in turn may have a detrimental impact on adolescents’ sleep [43]. Thus, poor sleep hygiene practices can be seen as potentially modifiable risk factor that may moderate some of the above-mentioned influences on adolescents’ sleep.

In this view, respecting sleep hygiene rules is an easy and cost-effective means to both maintain or re-install regular intra-individual sleep patterns. Specifically, sleep hygiene principles are defined as behavioral methods that promote sleep quality, adequate sleep time and complete consciousness during waking hours in a day [44]. Following LeBourgeois et al. [44], these practices include avoiding late-afternoon naps, alcohol, tobacco, and caffeine before bedtime, sleeping alone, not using the bed for activities other than sleep, sleeping in a comfortable, quite, toxin-free environment, maintaining a stable sleep schedule, following a bedtime routine, and avoiding bedtime activities that are physiologically, cognitively, and emotionally activating. Studies on students have revealed that respecting sleep hygiene rules is correlated with higher sleep quality and lower sleepiness during the day [45–49]. Nevertheless, some sleep hygiene domains seem to have a stronger impact on sleep quality and sleep duration than others [50]. For instance, while avoiding electronic devices before sleep has been suggested to be a useful strategy [51], little evidence exists that exercising in the evening has a negative impact on subsequent sleep [52]. In summary, these findings suggest that the validity of sleep hygiene recommendations should be examined more thoroughly in future research [50, 53]. Thus, having reliable and valid self-report measures is essential to gauge individual responses to behavioral interventions and evaluate sleep hygiene recommendations.

To assess sleep hygiene behavior, LeBourgeois et al. [44] have developed the Adolescent Sleep Hygiene Scale (ASHS). The ASHS is a self-report questionnaire specifically designed to examine sleep hygiene in 12- to 19-year-old adolescents, referring to sleep hygiene practices during the past month. More recently, Storfer-Isser et al. [54] have validated this instrument and developed a revised version (ASHSr), which contains six dimensions (physiological factor, behavior arousal factor, cognitive/emotional factor, daytime sleep factor, sleep environment factor, sleep stability factor), and which had improved psychometric properties, as described in more details in the methods section. The aim of the present study was to translate the English version into Farsi/Persian and to validate the Farsi/Persian version of the ASHSr. This endeavor is important because Iranian adolescents seem to share similar sleep pattern compared with adolescents for which the ASHSr was initially designed [55]. To this end, a large sample of Iranian adolescents (see below) completed a series of questionnaires. We evaluated the factorial validity of the ASHSr using confirmatory factor analyses. Moreover, Cronbach’s alphas for the overall ASHSr index and the various ASHSr subscales were calculated to examine the internal consistency of the instrument. Test-retest reliability was assessed with a subsample of students. To assess the concurrent validity of the ASHSr, participants also completed the Pittsburg Sleep Quality Index (PSQI).

## Methods

### Procedure

Three educational regions of Kermanshah (Iran) were randomly selected. The Kermanshah city consists of three educational regions. From each region, we randomly selected four elementary schools (two girls’ schools, two boys’ schools) and four high schools (two girls’ schools, two boys’ schools). Then, one class per grade was randomly selected from each of the 24 schools. Since there are three grades per school, a total of 72 classes were included.

Next, participants and their legal guardians were informed about the aims of the study, and that all data would be gathered anonymously. Both participants and their legal guardians signed the written informed consent. Participants completed self-rating questionnaires individually in their classroom during an official school lesson, covering socio-demographic and sleep-related information. To warrant data security, all participants put their completed questionnaires in sealed envelopes; thus, neither classmates nor teachers were able to know participants’ answers. To examine test-retest reliability, 203 randomly selected participants (20%) were randomly chosen to complete the ASHSr 6 weeks later once again (the second measurement took place during the same semester). The Review Board of the Kermanshah University of Medical Sciences (Kermanshah, Iran) approved the study, which was performed in accordance to the ethical principles described in the Declaration of Helsinki and its later amendments.

### Sample

The study population consisted of 1013 students, including 435 females (43%) and 578 males (57%). Mean age was 15.4 years (*SD* = 1.2; age range: 12–19 years). The initial sample consisted 1020 participants; however, seven participants had to be excluded from further data analysis due to incomplete data. At the moment when the data assessment took place all adolescents were free of acute illness/medication to ensure that their sleep habits are not influenced by these factors.

### Measures

#### Adolescent Sleep Hygiene Scale revised (ASHSr)

To obtain a Farsi/Persian version of the Adolescence Sleep Hygiene Scale (ASHS), we followed the algorithms proposed by Brislin [56]. First, two independent translators translated the English version into Farsi/Persian. Next, a third independent person compared the two translations, and, in case of differences, discussed the issues and performed the final draft. Next, two further independent translators performed the back-translation and compared the back-translated English versions with the original version. Finally, the final version mirrored the general agreement of all five researchers involved in this procedure.

The original ASHS is a 32-item self-report scale developed by LeBourgeois et al. [44], consisting of four qualitative items to ascertain usual bedtime and wake time on weekdays and weekends, and 28 quantitative items to assess nine domains of sleep hygiene practices. The domains include physiological (9 items), cognitive (6 items), emotional (3 items), sleep environment (4 items), daytime sleep (1 item), substances (2 items), sleep stability (4 items), bed time routine (1 item) and bed sharing (2 items) factors. All quantitative items assess sleep-facilitating and sleep-inhibiting practices, and are answered on a 6-point rating scale, referring to the past month, with the following response options: never (1), once in a while (2), sometimes (3), quite often (4), frequently-if not always (5), and always (6). Mean domain scores and an overall sleep-hygiene scores (mean of nine domain scores) can be calculated, with higher scores reflecting better sleep hygiene. LeBourgeois et al. (2005) initially tested the validity of the ASHS with 776 Italian and 572 American adolescents (12 to 17 years old, 655 males and 693 females). Taken together, moderate-to-strong linear relationships were found between sleep hygiene practices and sleep quality in both samples. Nevertheless, the findings of their study must be interpreted with caution because the nine different sleep hygiene domains were derived on a theoretical basis, without using exploratory factor analysis to test whether the items loaded adequately on the presumed factors. While the Cronbach’s alpha of .80 for the ASHS total scale was satisfactory, the internal consistency of several domains was below recommended levels [57], with Cronbach’s alphas varying between .37 and .74. To address these issues, Storfer-Isser et al. [54] thoroughly reexamined the psychometric properties of the ASHS with a sample of 514 adolescents (16 to 19 years) and revised the instrument to improve its psychometric properties. Based on the comments from the adolescents participating in the LeBourgeois et al. (2005) study, two items were added with regard to daytime sleep and sleep stability, whereas one domain (bed/bedroom sharing), which consisted of two items, was omitted. A first confirmatory factor analysis (CFA) model based on the remaining eight domains did not result into a satisfactory solution. Therefore, the substance abuse factor was deleted because the two items that comprised it were highly skewed. Next, a 7-factor model was tested based on the remaining 26 items. While this model converged, it still did not achieve adequate model fit. Storfer-Isser et al. (2013) therefore excluded one item with a low factor loading, omitted the bedtime routine factor (1 item), and combined the two (separate) cognitive and emotional factors into a single factor. Next, a 5-factor, 24-item model was tested; however, the model fit remained unsatisfactory because three items from the cognitive/emotional factor had low factor loadings. The model was therefore re-estimated, with these three items loading on a separate (new) “behavioral arousal” factor. This model generally had acceptable model fit, Root Mean Square Error of Approximation (RMSEA) = .07, 90% Confidence Interval (CI) = .06–.07, Tucker Lewis Index (TLI) = .93, with inter-factor correlations ranging between *r* = .15 and .64, *p* < .01. Finally, a second-order CFA was performed to find out whether an overarching sleep hygiene construct can be calculated. The CFA showed that between 13% and 76% of the variance in the sleep domains could be explained by the higher-order hygiene construct, with the theoretical model fitting well with the empirical data, RMSEA = .07, 90% CI = .06–.07, TLI = .93. In support of concurrent validity, the ASHSr total sleep hygiene score was significantly associated with longer objectively assessed sleep duration (*r* = .16), less night-to-night variability in sleep duration (*r* = −.21), higher sleep efficiency (*r* = .12), earlier bedtime (*r* = .17), and shorter sleep onset latency (*r* = .14). Moreover, a significant negative relationship was found with daytime sleepiness (*r* = −.26). Finally, evidence of convergent validity was found in the sense that the ASHSr overall scale was significantly associated with lower behavioral problems (*r* = −.18) and higher school competency (*r* = .25).

For the purpose of the present study, participants responded to the 24 quantitative items comprising the revised ASHS (ASHSr). As for the original scale, answers were given on a 6-point ordinal scale ranging from 1 (never) to 6 (always). Before calculating the total ASHSr and subscale scores, all items were reverse-coded so that higher values indicate better sleep hygiene practices.

#### Pittsburgh Sleep Quality Index (PSQI)

The Pittsburgh Sleep Quality Index (PSQI) was designed by Buysse et al. [58] to measure sleep quality and to help diagnose people with good or bad sleep. The PSQI is a self-report scale that is completed in 5 min: it consists of 19 items and contains seven subscales (subjective sleep quality, sleep latency, sleep duration, sleep efficiency, sleep disturbance, sleeping medication, daytime dysfunction), each equally weighted on a scale from 0 to 3, with higher scores indicating worse sleep quality. The seven components are then summed up to obtain an overall PSQI score, ranging from 0 (good sleep quality) to 21 (poor sleep quality). Total scores of > 5 reflect poor sleep, associated with considerable sleep complaints. In line with previous studies, the algorithm for sleep duration was specifically adjusted to young people [59, 60]. Responses were coded as follows: ≥ 8 h = 0, < 8 and ≥ 7 h = 1, < 7 and ≥ 6 h = 2, and < 6 h = 3. The Cronbach’s alpha for internal consistency was 0.83 in the initial validation study [58]. The psychometric properties of the Farsi/Persian version were tested by Farrahi et al. [61] in an adult sample of psychiatric patients and healthy controls. In their study, the Cronbach’s alpha level was 0.77 [61]. Furthermore, the instrument also had acceptable internal consistency (Cronbach’s alpha = .83) in a previous study with 2257 Iranian adolescents [62]. Answers are given on 4-points-Likert scales ranging from 0 (not at all or similar) to 3 (completely true/or similar), with higher scores reflecting a more impaired sleep.

### Statistical analysis

First, means, standard deviations, kurtosis and skewness were calculated for each ASHSr item as indicators of descriptive statistics. Next, a first-order CFA was carried out to test factorial validity of the ASHSr. We used maximum likelihood (ML) for parameter estimation, and considered multiple goodness-of-fit indices to find out whether the theoretical model fitted well with the empirical data [63]. Based on the recommendations of Byrne [64], model fit was considered adequate if the Adjusted Goodness of Fit Index (AGFI) was ≥ .90, Normed Fit Index (NFI) was ≥ .90, Comparative Fit Index (CFI) was ≥ .90, TLI was ≥ .90, Root Mean Residual (RMR) was ≤ .05, and if the RMSEA was ≤ .05. Following Comrey and Lee [65], standardized factor loadings of ≥ .71 were considered as excellent, ≥ .63 as very good, ≥ .55 as good, and ≥ .45 as fair. In a second step, a second-order CFA was performed to examine the extent to which an overarching sleep hygiene construct explained variance in each ASHSr factor. Cronbach’s alphas were then calculated to test internal consistency. Product-moment correlations were used to examine inter-factor correlations, as well as associations between the six domain-related ASHSr subscales and the ASHSr total sleep hygiene scale. Finally, product-moment correlations were used to examine test-retest reliability and concurrent validity (correlations between the ASHSr total and domain scales and the PSQI). Significant correlations of *r* < .30 are interpreted as small, of *r* = .30 to .50 as medium, and of *r* > .50 as large [66]. CFA was carried out with AMOS® 22 (IBM Corporation, Armonk NY, USA), all other analyses were done with SPSS® 23 (IBM Corporation, Armonk NY, USA).

## Results

### Descriptive statistics

Table 1 contains the descriptive statistics for all ASHSr items. Six items exceeded the theoretical mean score of *M* = 3.5, whereas 18 items were below this threshold. Table 1 also shows that according to the standards defined by West, Finch, and Curran [67], skewness and kurtosis were within acceptable limits (skewness < 2, kurtosis < 7) for all ASHSr items. Table 2 provides the descriptive statistics for the ASHSr overall index and subscales, whereas the descriptive statistics for the PSQI overall index and subscales are shown in Table 3. In the present sample, 63% (*n* = 638) of the adolescents were classified as poor sleepers (with PSQI overall scores >5).

**Table 1:** Descriptive statistics and standardized factor loadings from the first-order confirmatory factor analysis of the 24-item revised Adolescent Sleep Hygiene Scale (ASHSr)

	*M*	*SD*	Range	Skew	Kurt	Λ
Physiological Factor						
After 6:00 pm, I have drinks with caffeine (e.g., cola, pop, root beer, iced tea, coffee).	3.53	0.55	1–5	0.12	−1.37	.57^***^
During the hour before bedtime, I am very active (e.g., playing outside, running wrestling).	3.21	0.53	1–5	0.09	−1.33	.41^**^
During the hour before bedtime, I drink >4 glasses of water (or some other liquid).	3.67	0.51	1–5	−0.06	−1.14	.16^*^
I go to bed with a stomachache.	2.09	0.48	1–5	1.22	0.27	.68^***^
I go to bed feeling hungry.	2.11	0.45	1–5	1.15	0.27	.61^***^
Behavioral Arousal Factor						
During the hour before bedtime, I do things that make me feel very awake (e.g., playing video games, watching TV, talking on the telephone).	3.28	0.50	1–5	0.18	−1.04	.60^***^
I go to bed and do things in my bed that keep me awake (e.g., watching TV, reading).	3.11	0.51	1–5	0.24	−1.14	.70^***^
I use my bed for things other than sleep (e.g., talking on the telephone, watching TV, playing video games, doing homework).	2.97	0.53	1–5	0.40	−1.10	.70^***^
Cognitive/Emotional Factor						
I go to bed and think about things I need to do.	4.33	0.48	1–5	−0.54	−0.78	.19^*^
I go to bed and replay the day’s events over and over in my mind.	3.95	0.51	1–5	−0.23	−1.17	.29^**^
I check my clock several times during the night.	2.89	0.52	1–5	0.50	−0.92	.43^**^
During the 1 h before bedtime, things happen that make me feel strong emotions (sadness, anger, excitement).	3.27	0.53	1–5	0.24	−1.19	.51^***^
I go to bed feeling upset.	2.99	0.52	1–5	0.56	−0.94	.79^***^
I go to bed and worry about things at home or at school.	3.38	0.52	1–5	0.18	−1.19	.67^***^
Daytime Sleep Factor						
During the day, I take a nap that lasts >1 h.	3.16	0.56	1–5	0.26	−1.28	.84^***^
After 6:00 pm, I take a nap.	2.01	0.45	1–5	1.44	1.15	.39^**^
Sleep Environment Factor						
I fall asleep while listening to loud music.	2.99	0.56	1–5	0.33	−1.30	.51^***^
I fall asleep while watching TV.	2.99	0.52	1–5	0.32	−1.09	.59^***^
I fall asleep in a brightly lit room (e.g., the overhead light is on).	2.34	0.50	1–5	0.92	−0.40	.56^***^
I fall asleep in a room that feels too hot or too cold.	2.73	0.54	1–5	0.56	−1.06	.56^***^
I fall asleep in one place and then move to another place during the night.	2.22	0.49	1–5	1.05	−0.15	.57^***^
Sleep Stability Factor						
During the school week, I stay up more than 1 h past my usual bedtime.	3.34	0.52	1–5	0.12	−1.24	.36^**^
On weekends, I stay up more than 1 h past my usual bedtime.	4.20	0.51	1–5	−0.53	−0.90	.83^***^
On weekends, I sleep in more than 1 h past my usual wake time.	4.21	0.51	1–5	−0.50	−0.92	.69^***^

*Note*. Λ=Standardized factor loadings. Response choices were on a 6-point ordinal scale: 1 = never, 2 = once in a while, 3 = sometimes, 4 = quite often, 5 = frequently, if not always, 6 = always

^*^ *p* < .05. ^**^ *p* < .01. ^***^ *p* < .001

**Table 2:** Cronbach’s alpha values, test-retest reliabilities and inter-factor correlations from the first-order confirmatory factor analysis

	*M*	*SD*	1.	2.	3.	4.	5.	6.	7.
1. Physiological (α=.73)	4.08	1.01	(.87^***^)						
2. Behavioral Arousal (α=.74)	3.89	1.30	.34^***^	(.85^***^)					
3. Cognitive/Emotional (α=.71)	3.54	1.01	.34^***^	.47^***^	(.86^***^)				
4. Sleep Environment (α=.71)	4.35	1.12	.57^***^	.40^***^	.33^***^	(.84^***^)			
5. Sleep Stability (α=.75)	3.09	1.23	.16^**^	.32^***^	.32^***^	.30^***^	(.82^***^)		
6. Daytime Sleep (α=.75)	4.42	1.31	.34^***^	.44^***^	.31^***^	.35^***^	.28^***^	(.83^***^)	
7. ASHSr Total Score (α=.74)	3.88	0.77	.71^***^	.69^***^	.73^***^	.56^***^	.76^***^	.55^***^	(.85^***^)

Note*.* α=Cronbach’s alpha. Higher scores in the ASHSr reflect better sleep hygiene practices. In brackets, in the diagonale = Test-retest reliability coefficients for the six ASHSr subscales and the ASHSr total score

^**^ *p* < .01. ^***^ *p* < .001

**Table 3:** Correlations between the revised Adolescent Sleep Hygiene Scale (ASHSr) and the Pittsburgh Sleep Quality Index (PSQI)

	*M*	*SD*	Physiological	Behavioral Arousal	Cognitive/Emotional	Sleep Environment	Sleep Stability	Daytime Sleep	Total ASHSr
Subjective Sleep Quality	0.70	1.06	−.10^**^	−.14^**^	−.17^**^	−.06	−.12^**^	−.16^**^	−.18^**^
Sleep Latency	1.24	0.93	−.11^***^	−.06	−.08^*^	−.09^**^	.05	.08^*^	.00
Sleep Duration	0.99	0.93	−.30^***^	−.03	−.06*	−.06	−.06	−.06	−.10**
Sleep Efficiency	0.89	1.07	−.28^***^	−.05	−.01	−.28^***^	.02	−.09^**^	−.18^***^
Sleep Disturbance	1.39	0.66	.04	−.11^***^	−.12^***^	.01	−.09^**^	−.10^***^	−.08^**^
Sleep Medication	0.84	1.04	−.12^***^	−.16^***^	−.08^**^	−.15^***^	−.07^*^	−.11^***^	−.17^***^
Daytime Dysfunction	1.23	0.69	.05	−.06^*^	.00	.03	−.05	−.01	.00
Total PSQI index	7.30	3.69	−.22^***^	−.15***	−.11**	−.04	−.11***	−.10**	−.13***

Note. Higher scores in the ASHSr reflect better sleep hygiene practices. Higher scores in the PSQI indices represent lower sleep quality. Therefore, negative correlations indicate that better sleep hygiene practices are associated with better quality of sleep

^*^ *p* < .05. ^**^ *p* < .01. ^***^ *p* < .001

### Factorial validity of the ASHSr

#### First-order CFA

The 6-factor, 24-item first-order CFA suggested an adequate fit between the theoretical model and the empirical data, χ^2^/df = 2.99, AGFI = .91, NFI = .90, CFI = .93, CFI = .91 TLI = .91. RMR = .03, RMSEA = .05 (.04, .05). As shown in Table 1, the factor loadings of 15 items were good (Λ ≥ .55), and of six items fair (Λ ≥ .32). The factor loadings of three items remained below the recommended level of Λ ≥ .32. One of these items (“During the 1 hour before bedtime, I drink > 4 glasses of water or some other liquid.”) belonged to the physiological factor, whereas two items (“I go to bed and think about things I need to do.” “I got to bed and replay the day’s events over and over in my mind.”) belonged to the cognitive/emotional factor. Nevertheless, we decided not to delete these items for four reasons: First, the overall model fit of the CFA was acceptable. Second, Cronbach’s alpha values of the respective factors would not have increased after deletion of these items. Third, as shown in Table 2, the Cronbach’s alpha values of all ASHSr subscales exceeded the recommended levels of α ≥ .70 [57]. Fourth, retaining the items ensure comparability of Farsi/Persian version of the ASHSr and the English original, which might be important for cross-cultural comparisons.

The mean scores of the six ASHSr subscales and the ASHSr total sleep hygiene scale is presented in Table 2. Table 2 also displays the inter-factor correlations based on the first-order CFA. Inter-factor correlations varied between Ψ = .16 (physiological and sleep stability factor) and Ψ = .57 (physiological and sleep environment factor), suggesting adequate convergent and divergent validity of the ASHSr.

#### Second-order CFA

**Fig. 1:**
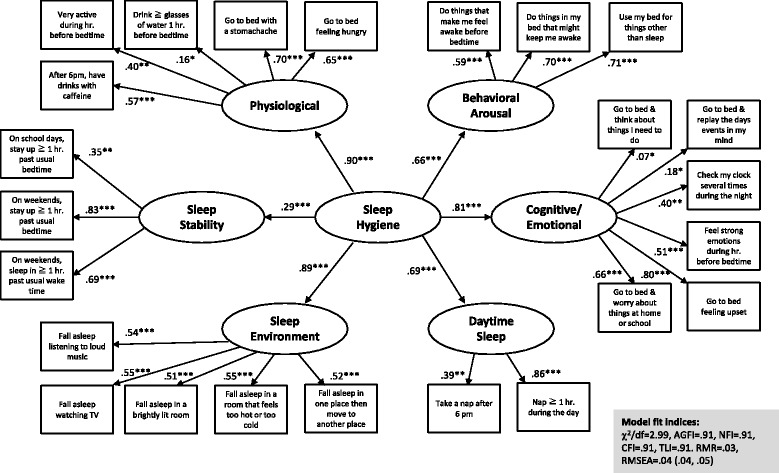
Factor structure of the revised 24-item Adolescent Sleep Hygiene Scale (ASHSr) and factor loadings from the second-order confirmatory factor analysis

The findings of the second-order CFA are shown in Fig. 1. The model fit, χ^2^/df = 2.99, AGFI = .91, NFI = .91, CFI = .91, CFI = .91 TLI = .91. RMR = .03, RMSEA = .04 (.04, .05), was very similar to the one of the first-order CFA. This also applied to the factor loadings. Again, three items loaded relatively weakly on the respective factors, but were retained in the model for the reasons described above. The γ coefficients between the second-order construct and the six factors ranged between γ = .29 (sleep stability factor) and γ = .90 (physiological factor), indicating that the sleep hygiene construct explained between 8.4% and 81% of variance in the sleep stability factor and the physiological factor, respectively.

#### Internal consistency reliability

The internal consistency reliability was satisfactory for the ASHSr total sleep hygiene scale (α = .85), and for all ASHSr subscales, with Cronbach’s alphas for the specific domains ranging from .82 to .87.

#### Test-retest reliability

Table 2 shows that the test-retest reliability was *r* = .85 for the ASHSr total sleep hygiene scale, whereas test-retest reliabilities ranged between *r* = .82 (sleep stability factor) and *r* = .87 (physiological factor) for the specific ASHSr domains.

#### Concurrent validity

Table 3 shows that the ASHSr total sleep hygiene scale and the ASHSr subscales were statistically significantly, but weakly correlated with the sleep quality measures assessed with the PSQI. A significant association was found between the ASHSr total sleep hygiene index and the PSQI overall index (*r* = −.13, *p* < .001), indicating that better sleep hygiene practices are associated with better overall sleep quality.

## Discussion

The present study lends support to the 6-factor structure of the Farsi/Persian version of the ASHSr, confirms that all indices have satisfactory internal consistency, and shows that the ASHSr has adequate test-retest reliability. While significant correlations were found between the ASHSr and the PSQI (in support of concurrent validity of the instrument), the magnitude of the relationships was weak. Our findings add to the literature regarding the association between adolescents sleep hygiene practices and their sleep quality, which is still a relatively under-researched area. Developing instruments to assess sleep hygiene practices among Iranian adolescents is important because sleep complaints are highly prevalent in this target group. For instance, in the present sample, the percentage of students with low sleep quality was 63%, which is higher compared to previous studies with adolescents from New Zealand [59] or Iran [62], in which just over half of the participants were classified as poor sleepers.

The 6-factor structure found in the CFA is in line with a prior study with American adolescents [54]. Compared to this study, three items had relatively low factor loadings. However, we decided to retain these items to ensure comparability with other studies, and because these items seemed clinically relevant. For instance, going to bed and think about things that need to be done and/or replaying the day’s events in one’s mind are representative of dysfunctional sleep-related cognitions, and therefore key elements of cognitive models of insomnia [68, 69]. Not surprisingly, rumination and focusing proved to be associated with increased sleep complaints in previous investigations [70]. Accordingly, these items might play an important role in sleep hygiene planning with adolescent samples. Since it is not fully clear why these items had low factor loadings, more research is needed to corroborate the results found in the present study.

Compared to the study of Storfer-Isser et al. [54], the inspection of the single item descriptive statistics showed that Iranian adolescents reported considerable worse sleep hygiene practices than American youngsters, with Iranians having less favorable scores on 20 of 24 items. This is in line with a recent study in a nationwide sample of New Zealand adolescents reporting more favorable scores on the ASHS total index, and four subscales (physiological, cognitive/emotional, sleep environment, daytime sleep) [59]. In the present sample of Iranian adolescents, the most frequently reported problematic sleep hygiene practices (exceeding the theoretical mean of 3.5) were (a) going to bed and thinking about things that need to be done, (b) going to bed an replaying the day’s events over and over in mind, (c) staying up longer and (d) “sleeping in” more than 1 h than usual bedtime/wake time during weekends, (e) drinking > 4 glasses of water/liquid 1 h before bedtime, and (f) drinking caffeine (e.g., cola pop, root bear, iced tea, coffee) after 6:00 pm. Thus, these issues seem to be the most important aspects of sleep hygiene that could be addressed in future intervention programmes.

Compared to American and New Zealand adolescents, better sleep practices were reported by Iranian adolescents with regard to the behavioral arousal factor, indicating that before bedtime, Iranian adolescents less frequently engage in activities that make them feel awake (e.g., playing video games, watching TV, talking on the telephone). Cultural differences with regard to sleep hygiene have been reported previously. For instance, LeBourgeois et al. [44] found that Italian adolescents reported better sleep hygiene practices than American peers, which they explained by a stronger parental involvement into adolescence among Italian youngsters. To what extent parental involvement impacted on sleep hygiene practices among Iranian adolescents cannot be directly answered with the present data. However, at least two lines of research on sleep in Iranian children and adolescents suggest that parental involvement on adolescents’ sleep patterns is important: First, a sleep hygiene training for both parents and children with ADHD improved children’s sleep and psychological functioning [36]. Second, from a former study [35] on sleep and psychological functioning among 81 families in North-eastern Iran, it turned out that parents’ and children’s sleep patterns and psychological functioning were similar; importantly, this pattern of results was in line with previous research on sleep and psychological functioning in Swiss families [34]. Given the obvious differences in sleep hygiene practices between adolescents from different cultures, clearly more knowledge is needed regarding the question of how psychological, social and environmental factors impact on sleep hygiene practices of adolescents in Western and non-Western societies.

With regard to the psychometric properties of the Farsi/Persian version of the ASHSr, we found higher Cronbach’s alpha values compared to previous research with American and Italian adolescents [44, 54]. Moreover, despite the fact that the recall period of the ASHSr (past month) and the time interval between the first and the second measurement occasion (6 weeks) did not perfectly match in the present study, our findings show that the ASHSr indices have adequate test-retest reliability with strong correlations across a 6-week period.

With regard to concurrent validity, our findings are in line with previous studies using the ASHS/ASHSr showing that sleep hygiene indices are significantly associated with other subjective and objective sleep quality measures [44, 54, 71–73] or in which worse sleep hygiene practices were reported by adolescents with insomnia compared to normal sleepers [73]. Nevertheless, as in a previous study using actigraphy-based sleep outcomes (e.g. sleep duration, sleep efficiency, sleep onset latency) and self-reported daytime sleepiness [54, 74], the strength of the relationships found between the ASHS instruments and other sleep indices was relatively weak (*r* ≈ .20). The reasons for the low correlations are not fully understood, particularly as higher correlations were found between the ASHS and the PSQI among New Zealand adolescents [59]. While the PSQI may not be an age-appropriate instrument to assess sleep quality in adolescents (e.g., the instrument does not measure the possible discrepancy between weekday and weekend sleep parameters) [74], another explanation for the low correlations might be that sleep hygiene practices were relatively poor in the present sample, resulting in more limited variance compared to other samples. For instance, as reported in Table 1, only six ASHSr items were above the theoretical mean of *M* = 3.5, whereas the remaining 18 items were below this threshold. Thus, there seems to be considerable scope for improvement with regard to sleep hygiene among Iranian adolescents. Hence, it would be interesting to see whether targeting healthy sleep hygiene practices among adolescents results in stronger correlations between the ASHSr and sleep outcomes in this specific target group [49].

Some limitations need to be acknowledged that preclude an overgeneralization of the present findings: First, all data are based on self-reports. However, self-report questionnaires are still useful for large-sample research as time- and cost-effective tools. Second, testing the discriminative validity of the ASHSr was not possible because the findings are based on a non-clinical sample of adolescents. Third, convenience sampling was used to select participants. Therefore, the sample may not be fully representative of the entire Iranian student population. For instance, male students were slightly overrepresented in the present sample (57% boys vs. 43% girls). Moreover, a generalization to other age groups is not possible. Fourth, while we used the PSQI to assess concurrent validity, clinical interviews or other instruments such as the Insomnia Severity Index [75, 76], which provide validated cut-off scores for subthreshold/moderate/severe insomnia, might have been more useful to relate the ASHSr with sleep complaints. Nevertheless, among adolescents, valid and reliable instruments to measure sleep hygiene practices are highly needed since adolescence is a period in which considerable changes in sleep/wake pattern, sleep duration, delay in the timing of sleep, and increasingly large discrepancies between weekday and weekend sleep pattern occur [28, 44, 54]. Moreover, while many adolescents have limited awareness that sleep hygiene practices are closely associated with sleep quality [49, 77–79], research suggests that sleep hygiene practices can be improved through adequate training [46, 48, 80, 81]. Fourth, more evidence is needed with regard to the predictive validity of the ASHSr, which was not tested in the present study.

## Conclusion

The findings showed that the Persian/Farsi version of the ASHSr has acceptable validity and reliability among Iranian adolescents. In summary, the ASHSr offers a comprehensive, time- and cost-effective tool to examine various sleep-hygiene domains among adolescents. Better understanding adolescents’ sleep hygiene is an important prerequisite to optimize programs to educate young people about good sleep habits. The ASHSr might be particularly useful for screening, counseling purposes, and for the evaluation of interventions targeting sleep hygiene. For instance, the ASHSr may help caretakers to ensure that adolescents have regular sleep/wake schedules, healthy sleep environments, and suitable bed routines helpful to prepare them emotionally, cognitively and behaviorally for a good night sleep.

## Abbreviations

AGFI: Adjusted goodness of fit index
ASHS: Adolescent sleep hygiene scale
ASHSr: Revised version of the adolescent sleep hygiene scale
CFA: Confirmatory factor analysis
CFI: Comparative fit index
CI: Confidence interval
ML: Maximum likelihood
NFI: Normed fit index
PSQI: Pittsburgh sleep quality index
RMSEA: Root mean square error of approximation
TLI: Tucker lewis index
